# Supplementary material for: Lessons from an International Initiative to Set and Share Good Practice on Human Health in Environmental Impact Assessment
Source: Int J Environ Res Public Health. 2021 Feb 3;18(4):1392. doi: 10.3390/ijerph18041392 (PMC7913344; doi:10.3390/ijerph18041392)
Supplement: Supplementary file 1 [file ijerph-18-01392-s001.pdf]

Supplementary Materials File S1: Supplementary information

Lessons from an International Initiative to Set and Share Good Practice on Human Health in Environmental Impact Assessment

Ben Cave [1,2,3,4,*], Ryngan Pyper [1,2], Birgitte Fischer-Bonde [1,2,5], Sarah Humboldt-Dachroeden [2,6] and Piedad Martin-Olmedo [3,7,8]

[1] BCA Insight Ireland Ltd., D02FY24 Dublin, Ireland; ryngan.pyper@bcainsight.com (R.P.); birgittefischerbonde@gmail.com (B.F.-B.)
[2] International Association for Impact Assessment (IAIA), Fargo, ND 58103-3705, USA; sarahhumboldt92@gmail.com
[3] European Public Health Association (EUPHA), Post Box 1568, 3500 BN Utrecht, The Netherlands; piedad.martin.easp@juntadeandalucia.es
[4] Centre for Primary Health Care and Equity, University of New South Wales, NSW 2052 Sydney, Australia
[5] Fischer-Bonde Consulting, 1727 Copenhagen, Denmark
[6] Department of Social Science and Business, Roskilde University, 4000 Roskilde, Denmark
[7] Escuela Andaluza de Salud Publica, 18011 Granada, Spain
[8] Instituto de Investigacion Biosanitaria de Granada (Ibs. GRANADA), 18016 Granada. Spain
* Correspondence: ben.cave@bcainsight.com

1. Methods

The findings from the consultation events have been analysed in different ways. Participants in the technical meeting and the webinar provided comments from a general perspective i.e. they were not reviewing a written document. In the technical meeting, participants had prior sight of the first draft but comments were drawn from plenary and small workshop discussions. Participants in the webinar were invited to send questions beforehand and put questions to the presenters through the chat function during the webinar. The responses given to the survey and the peer review comments were each based upon a reading of the second and third drafts, respectively, of the reference paper. These comments were analysed with the themes identified by Herber et al. [1]. This framework relates to peer review of a qualitative manuscript and enables analysis by theme rather than reporting criteria.

1.1. Technical meeting

The technical meeting discussed a first draft of a reference paper which set out high-level principles for covering population and human health in EIA with a focus on the requirements of the EIA Directive (2011/92/EU as amended by 2014/52/EU [2]. The participants included Environment and Health Focal Points from Member States within the WHO European Region plus officers of the European Commission, the European Investment Bank, universities, health and environment institutes as well as environmental consultancies.

There were plenary sessions at the start and end of each day. Participants also worked in three groups to discuss these questions. The information and notes from presentations and discussions were used to prepare a report. A revised reference paper was issued as a consultation draft.

1.2. Webinar

The consultation (second) draft of the reference paper was presented and discussed at a webinar hosted by IAIA (3rd December 2019). 217 participants from 30 countries took part. The target audience included practitioners conducting EIA, researchers involved in EIA or HIA, developers and authorities requested to express their opinion on the information supplied in an EIA report. The webinar included three presentations which covered the aim of the paper, a definition of EIA, the main changes introduced in the

amended EIA Directive, EIA and human health, principles for human health in EIA and the EIA process. Attendees were able to ask questions. They were also invited to send comments and feedback.

A thematic coding strategy was applied to identify common themes for the analysis of those comments, and results presented according to them. The categories used were in line with the specific key changes of concern included in the amended EIA Directive mentioned previously as specific objectives.

1.3. *Online survey*

The online survey was conducted on the consultation (second) draft of the reference paper. The survey ran from 14th November 2019 to the 6th of January 2020. It was hosted on SurveyMonkey and advertised via social media (LinkedIn, Twitter) and on the websites of IAIA and EUPHA. The survey was anonymous and consisted of both open and closed questions (full questionnaire available as Supplementary material).

Questions 1-4 addressed demographic information of the participants and questions 5-8 inquired about the clarity and structure of the document as well as any missing concepts. The questionnaire then enquires about the clarity and comprehensiveness of each chapter. Participants were invited to agree or disagree with the proposed issue (Options: Yes / No / Don't know), and there was open space to add information that either was missing or not clear in the document.

The same thematic coding used for inputs from the Webinar was applied to the qualitative information from the survey to identify issues relevant to improving drafting the position document.

The survey comments were analysed with the themes identified by Herber et al. [1]. This framework is considered appropriate as it relates to peer review of a qualitative manuscript and enables analysis by theme rather than reporting criteria. The latter, e.g. COREQ [3] or SRQR [4], were considered, but were not deemed suitable as an analysis framework for peer review comments because the manuscript is a resource document rather than a study.

1.4. *Peer review*

The peer reviewers were selected by IAIA for their expertise in public health, environmental assessment and knowledge of the EIA Directive. The review was conducted anonymously although one reviewer revealed their identity in the course of the review. The identity of the reviewers is given in the reference paper.

The peer review comments were also analysed with the themes identified by Herber et al. [1]. The analysis was conducted for each reviewer but the results are combined in this paper to preserve anonymity.

1.5 *. Other*

In addition to these events the second draft of the reference paper was presented and discussed at the conference for the European Public Health Association (Marseilles, November 2019) [5], and a work in progress was presented at an IAIA symposium (online, September 2020).

2. Results

2.1. *Technical meeting*

Three workshops facilitating discussions around the proposed position paper were held. Concrete feedback was accumulated, challenges and opportunities identified as well as how these could be used to update the draft position paper. The results of the workshop were divided into the section of the draft position paper. **Error! Reference source not found.** lists these dimensions and gives an overview of which issues were addressed in the position paper.

Table S1. Key changes identified through technical meeting.

Copyright: © 2021 by the authors. Licensee MDPI, Basel, Switzerland. This article is an open access article distributed under the terms and conditions of the Creative Commons Attribution (CC BY) license (http://creativecommons.org/licenses/by/4.0/).

Dimensions	Specific observations to be addressed
How should population and human health be defined in EIA?	Human Health: Use the WHO definition. Both components of this definition are important. All determinants of health.<br>Population health:<br>- Baseline health conditions including demographic profiles<br>- Definition of vulnerable groups and vulnerability
What is a significant effect for population and human health in EIA?	Transparency in decision-making processes to ensure that determination of effects that are likely and significant is based on professional judgement and good evidence.<br>Assessment is at population level.<br>Consider severity of outcomes for human health<br>Ensure that the determination of significance is also informed by: local and national health priorities; input from health professionals (public health) and public engagement.
How should changes in health be reported in EIA?	Engage public health experts.<br>Engage stakeholders and the public<br>Reporting must use best available scientific evidence<br>Consider cumulative effects.<br>Data must be as precise as possible.<br>Provide a model/framework that spans all determinants of health.<br>The DPSEEA framework can be used as a health pathway model [6]<br>Define health outcomes and use health indicators<br>Consider different contexts<br>Where possible and when proportionate, establish monitoring to track health outcomes (nb monitor significant effects).
What counts as evidence for changes in health?	Scientific and peer-reviewed literature<br>Additional sources for evidence of health changes: exposure scenario analysis, health risk assessments and project conditions based on the project proposal
What is the relationship between EIA and HIA?	EU-level legislative regulation for health in EIA but not for HIA
How should the health sector participate in the EIA process?	Engage health experts early<br>Involve a multidisciplinary team.<br>Ministry of Health can take an active role.<br>Consider coherence of the country's legislation and political background.<br>Health authority can provide input on health outcomes, pathways, effects on population health, follow-up and mitigation and monitoring.<br>National and regional health authorities have an important role in reviewing health chapters of EIAs.<br>Provide appropriate resources to engage health sector.<br>Provide training.

Copyright: © 2021 by the authors. Licensee MDPI, Basel, Switzerland. This article is an open access article distributed under the terms and conditions of the Creative Commons Attribution (CC BY) license (http://creativecommons.org/licenses/by/4.0/).

**Dimensions**	**Specific observations to be addressed**
What are the risks from a business-as-usual coverage of population and human health in EIA?	To avoid neglecting the health sector, health professionals from various disciplines must be engaged.<br>To enhance the detection of health implications and facilitate mutually agreed outcomes of the assessment, public engagement and participation must be fostered<br>To increase knowledge on health determinants, training possibilities must be provided
Who can conduct an assessment?	Expertise in public health needed. It is, at least, desirable to have a public health background with knowledge and skills across relevant health determinants.<br>Capacity building: enable and promote specialisation in impact assessment in training curricula of university studies on public health; mandatory training certificates should include health in EIAs; provide trainings and courses for professional development.<br>A team should have mixed skills, the ability to translate and adapt to different contexts.<br>Technical skills: for example, understanding of EIA process, legal and ethical requirements<br>Softer skills: for example, flexible attitude.

Analysing the comments and ideas of the meeting participants was helpful in mapping sections of the reference paper in need of further attention. Further, the discussion in the workshops assured that the general direction of the working group was going in the right direction.

2.2. *Webinar*

Participants attending the webinar formulated 73 comments, out of which 31 addressed relevant issues for improving the position paper (see **Error! Reference source not found.**). The remaining comments were appreciation for the event and the proposed document. Only a few also tried to bring attention towards other topics out of the scope of the position paper, such as the need to improve the health assessment within the Strategic Environmental Assessment or extending the equity assessment to other context different from EIA.

Table S2. Key changes identified through webinar.

**Dimensions**	**Specific observations to be addressed**
Target audience of the reference paper	Better description of the purposes of the document and main potential target users
Health Determinants	Gender perspective<br>Perceived health and risk perception<br>Definition of environment
Significance / likely health effect	Better description of how the significance of health impacts should be addressed.<br>Consider including the approach of combining magnitude of the effect and sensitivity of affected population in the characterization of the significance of health effects

**Dimensions**	**Specific observations to be addressed**
Equity / vulnerable groups	Integrating equity when addressing human health impacts<br>Make emphasis in addressing vulnerable groups
Methods / Tools	Consider the use of qualitative methods and data for addressing cultural beliefs of health and wellness<br>Differentiate between burden of diseases and health risks in characterising the significance of health impacts.<br>Address the characterization of health impacts related to cumulative exposure to multiple stressors.<br>Suggest generating good baseline health data for conducting HIA<br>Define indicators and thresholds for assessing human health<br>Add indications on the characterization of health impacts in resource limited situations/regions<br>Ensure proper health impacts characterization, not only as a cosmetic add-on<br>Add information on available software and computer applications for calculating health impacts adopted to specific sectors (e.g. power plant)<br>Add specific information referring to tools and techniques for assessing health effects and offset.<br>Introduce a section on communicating health impacts that help in presenting the variability of addressed impacts, and permutations among various HIA "axes.<br>Acknowledge other existing checklists<br>Acknowledge challenges for characterising health impacts at basic data quality level
Role of PH professionals / Competences needed	Acknowledge established PH competences sets (e.g. ASPHER)<br>Suggest the need of expertise in epidemiology<br>Specify possible roles of PH professionals throughout the EIA/HIA process<br>Involving experts with a PH degree should be a prerequisite for conducting EIA
Intersectoral cooperation / stakeholders / responsible authorities	Emphasise the need of bridging different interests<br>Take into consideration the demands from the community for characterizing impacts of new projects in the context of the whole affected area, especially those with multiple polluting sources.<br>Underline the need to build up intersectoral cooperation, if necessary, by promoting capacity building programmes<br>Engagement of different stakeholders<br>Suggest procedures for better collaboration between proponents and local authority

2.3. *Online survey*

In total, respondents to the survey provided 185 comments. These are summarised in **Error! Reference source not found.**.

64.5% of the respondents to the online survey were aged between 35 and 56 years old. 73% of respondents worked in public health, 47% in the environmental sector, 18% in social areas, 13% in medicine and 2% in other areas. Most respondents were employed in the public sector (44.5%) or within academia (38%), followed by 22% working in the private sector and 22% as independent consultants. Minorities of respondents were developers (6.7%) or project funders (2.5%). In total, 92% of the respondents reported that they worked mostly in EU countries, with 40% working in countries outside of the European Union.

86% reported that they found the position paper to be clear. **Error! Reference source not found.** shows the responses when respondents were asked about the clarity of specific chapters: the percentage of respondents stating that no further explanation was needed ranged from 41% (for 'expertise for conducting a health assessment within EIA') to 65% (for 'screening').

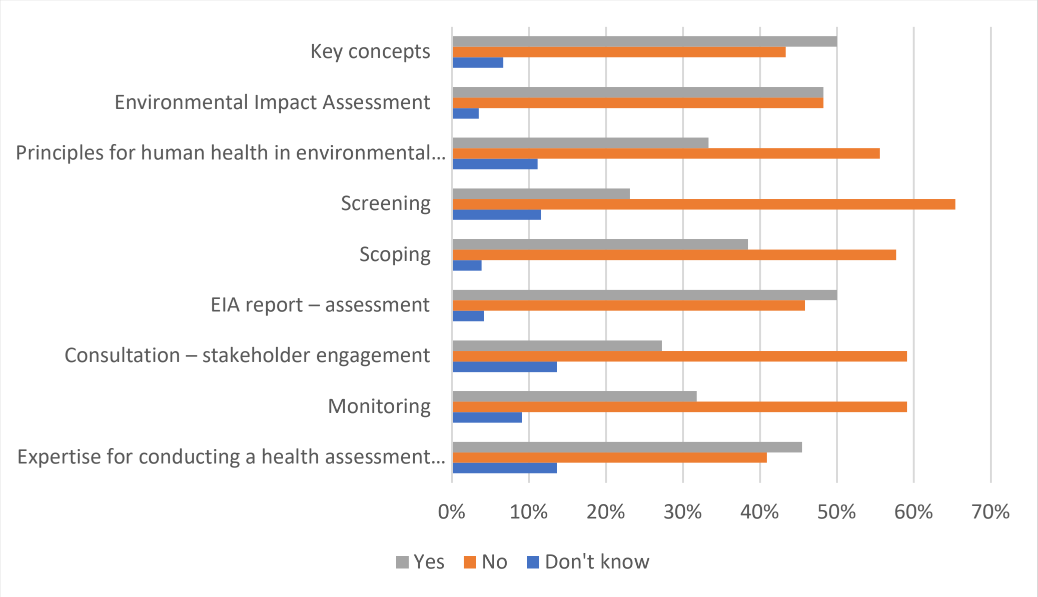

Figure S1. Areas that should be explained better, or additional areas, concepts or definitions that should be added?

Error! Reference source not found. shows that respondents found the good practice actions in each chapter to be clear, ranging from 70% (for 'expertise for conducting a health assessment within EIA') to 91% (for 'Monitoring'; 'Consultation – stakeholder engagement'; and 'EIA report – assessment'). NB section titles were edited in between versions.

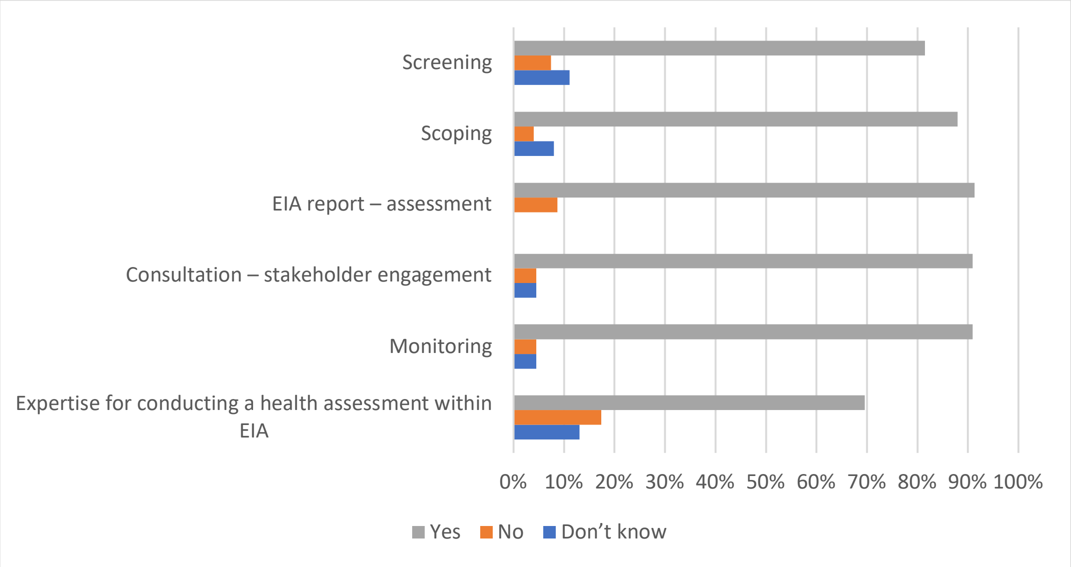

Figure S2. Are the 'good practice actions' presented in each chapter clear?

Table S3. Key changes identified through the survey.

Dimensions	Specific observations to be addressed
Target audience	Better definition of target audience in the introduction
Concepts	Introduce a distinction between risk factors and determinants of health under concept<br>Better differentiation between equity and health inequalities<br>Better clarification between population and human health
Health Determinants	Make reference to perceive health and risk perceptions as determinants of health to be considered when conducting screening and EIA report-assessment<br>Address specifically gender as a health determinant for evaluating vulnerability.
Significance / likely health effect	Elaborate criteria for establishing "significance effect"<br>Emphasise to assess positive and negative health impacts as well as environmental impacts<br>Introduce the concept of deprivation for establishing significance in EIA<br>Refer to pre-defined environmental criteria or thresholds set by national legislation when screening.<br>Relevancy of community concern in defining the significance of a health impact
Equity / vulnerable groups	Emphasise the influence of the interactions between upstream health determinants and inequalities in health outcomes<br>Elaborate on health within the principle of "equity"<br>Include section on inequalities and vulnerable groups
Methods / Tools	Stress that consistency of screening and assessment should be based on the 'best available evidence', recognising that often evidence may be missing for some impacts or casual pathways.<br>Add quantitative HIA methods<br>Consider cumulative effects from interactions and/or interventions.<br>Emphasise the need to look for quality available data both for characterising health determinants and health outcomes<br>Elaborate on early warning systems to potentially intervene during or after implementation and operation<br>Elaborate on the need for best available evidence for the principle of "consistency"
Role of PH professionals / Competences needed	Emphasise the need for early engagement of health experts at first stages of the EIA process, and even at starting the design of a project.<br>Make explicit the need for health practitioners to be trained on EIA.<br>Clarify expertise needed for conducting health assessment within EIA, and who should undertake health assessments within EIA<br>Add that expertise should go beyond environment and health. When necessary, include relevant experts e.g. chemists, engineers, social scientists
Intersectoral cooperation / stakeholders / responsible authorities	Highlight the need for involving intersectoral teams with experts from environment and health collaborating at all stages<br>Describe the purpose of the Consultation chapter more clearly and provide examples<br>Add to establishing sustainable feedback procedures with stakeholders<br>Reassess terminology for "public participation", "engagement" or "consultation"; using terms consistently

In line with Day et al. [7] a reviewer comment was defined as "*a distinct statement or idea found in a review, regardless of whether that statement was presented in isolation or was included in a paragraph that contained several statements.*" **Error! Reference source not found.** below sets out the thematic coding framework after Herber et al. [1] and then the counts for comments under each coded theme. Further columns indicate whether the comment was accepted, and edits made (Edit Y), if the comment was considered but no edit made (Edit N) and if the comment was noted but did not relate to a proposed edit (Edit NA).

Table S4. Type of comments made in survey.

Condensed theme codes	Theme/sub-theme	Count	Edit		
			Y	N	NA
A	Further information, clarification, explanation or justification needed	130	92	38	0
B	Confirmation/approval (from reviewer)	13	0	0	13
C	Miscellaneous	3	0	0	3
D	Structure	7	0	7	0
E	Re-wording, typos, proofing or readability edits	26	20	6	0
H	Implications for research/practice/theory/teaching etc.	6	2	4	0
	Total	185	114	55	16

Themes and sub-themes are adapted from Herber et al. [1].

2.4. *Peer review*

There were 210 comments between the three reviewers. Detailed tracked comments and overview statements were analysed.

Error! Reference source not found. below sets out the thematic coding framework after Herber et al. [1] and then the counts for comments under each coded theme. Further columns indicate whether the comment was accepted and edits made (Edit Y), if the comment was considered but no edit made (Edit N) and if the comment was noted but did not relate to a proposed edit (Edit NA).

Table S5. Type of comments made in peer review.

Condensed theme codes	Theme/sub-theme	Count	Edit		
			Y	N	NA
A	Further information, clarification, explanation or justification needed	54	42	11	1
B	Confirmation/approval (from reviewer)	16	1	0	15
C	Miscellaneous	6	1	0	5
D	Structure	8	2	3	3
E	Re-wording, typos, proofing or readability edits	66	49	15	2
F	Absence of important	2	1	1	0

Condensed theme codes	Theme/sub-theme	Count	Edit		
			Y	N	NA
	background information				
G	Inconsistency from EU EIA Directive	48	47	1	0
H	Implications for research/practice/theory/teaching etc.	10	7	3	0
I	Further literature references needed	0	0	0	0
	Total	210	150	34	26

Themes and sub-themes are adapted from Herber et al. [1].

References

1. Herber OR, et al. What feedback do reviewers give when reviewing qualitative manuscripts? A focused mapping review and synthesis. *BMC Med Res Methodol* 2020; **20**(1): 122. http://dx.doi.org/10.1186/s12874-020-01005-y
2. Directive 2014/52/EU of the European Parliament and of the Council amending Directive 2011/92/EU on the assessment of the effects of certain public and private projects on the environment. *Official Journal of the European Communities* 2014; **L 124**: 1-18. http://eur-lex.europa.eu/eli/dir/2014/52/oj
3. Booth A, et al. COREQ (Consolidated Criteria for Reporting Qualitative Studies). Guidelines for Reporting Health Research: A User's Manual; 2014: 214-26. https://onlinelibrary.wiley.com/doi/abs/10.1002/9781118715598.ch21
4. O'Brien BC, Harris IB, Beckman TJ, Reed DA, Cook DA. Standards for reporting qualitative research: a synthesis of recommendations. *Acad Med* 2014; **89**(9): 1245-51. http://dx.doi.org/10.1097/acm.0000000000000388
5. Cave B, et al. The amended EIA directive creates opportunities for public health. *European Journal of Public Health* 2019; **29**(Supplement_4). http://dx.doi.org/10.1093/eurpub/ckz185.541
6. Reis S, et al. Integrating health and environmental impact analysis. *Public Health* 2015; **129**(10): 1383-9. http://dx.doi.org/10.1016/j.puhe.2013.07.006
7. Day FC, Schriger DL, Todd C, Wears RL. The use of dedicated methodology and statistical reviewers for peer review: a content analysis of comments to authors made by methodology and regular reviewers. *Ann Emerg Med* 2002; **40**(3): 329-33. http://dx.doi.org/10.1067/mem.2002.127326
